# Monitoring and evaluation of COVID-19 response in the WHO African region: challenges and lessons learned

**DOI:** 10.1017/S0950268821000807

**Published:** 2021-04-14

**Authors:** Benido Impouma, Caitlin M. Wolfe, Franck Mboussou, Bridget Farham, Tessa Saturday, Cyril Pervilhac, Nsarhaza Bishikwabo, Tamayi Mlanda, Arish Bukhari Muhammad, Fleury Moussana, Ambrose Talisuna, Humphrey Karamagi, Olivia Keiser, Antoine Flahault, Joseph Cabore, Matshidiso Moeti

**Affiliations:** 1 World Health Organization, Regional Office for Africa, Brazzaville, Congo; 2 Institute of Global Health, University of Geneva, Geneva, Switzerland; 3 College of Public Health, University of South Florida, Tampa, Florida, USA

**Keywords:** African region, COVID-19, health emergencies, monitoring and evaluation

## Abstract

Monitoring and evaluation (M&E) is an essential component of public health emergency response. In the WHO African region (WHO AFRO), over 100 events are detected and responded to annually. Here we discuss the development of the M&E for COVID-19 that established a set of regional and country indicators for tracking the COVID-19 pandemic and response measures. An interdisciplinary task force used the 11 pillars of strategic preparedness and response to define a set of inputs, outputs, outcomes and impact indicators that were used to closely monitor and evaluate progress in the evolving COVID-19 response, with each pillar tailored to specific country needs. M&E data were submitted electronically and informed country profiles, detailed epidemiological reports, and situation reports. Further, 10 selected key performance indicators were tracked to monitor country progress through a bi-weekly progress scoring tool used to identify priority countries in need of additional support from WHO AFRO. Investment in M&E of health emergencies should be an integral part of efforts to strengthen national, regional and global capacities for early detection and response to threats to public health security. The development of an adaptable M&E framework for health emergencies must draw from the lessons learned throughout the COVID-19 response.

Recurring disease outbreaks characterise the 47 countries of the World Health Organization (WHO) African region, along with other public health events that threaten national and regional health security. Over 100 events are detected and responded to annually [1], with more than 348 detected across the region from 2017 to 2019. Preparedness and response to these events is guided by a set of standard protocols and procedures, which fall under the International Health Regulations (IHR) 2005 [2], informed by both the WHO Emergency Response Framework [3] and the Integrated Disease Surveillance and Response Framework (IDSR) [4]. These protocols require countries to build and maintain a set of core capacities that are essential for early detection and response to acute public health events in order to avoid interference with trade and travel, as was seen in the West African Ebola virus disease (EVD) outbreak in 2014–2016, and which is now the case with the ongoing COVID-19 pandemic in the region. COVID-19 is unique in that it has affected all 47 countries of the WHO African region simultaneously, requiring a robust multisectoral response, with the WHO Regional Office for Africa (WHO AFRO) overseeing the regional response.

Previous response to outbreaks has resulted in the development of a number of Strategic Preparedness and Response Plans (SPRP), all of which have contained a monitoring and evaluation (M&E) component, albeit often underdeveloped. WHO AFRO is responsible for providing support to all countries in their implementation of public health and social interventions in order to interrupt transmission, monitor trends and identify areas that require further support. Additionally, WHO AFRO prioritises interventions and resource allocation across all affected countries. The scale and magnitude of the COVID-19 pandemic in the region required that the M&E component of the COVID-19 SPRP be expanded and transformed into a stand-alone Monitoring and Evaluation Framework for COVID-19 in the WHO African region [5]. This was closely aligned with the WHO Monitoring and Evaluation Framework [6] and originated from the global 2019 COVID Strategic Preparedness and Response Plan [7], and the Strategic Response Plan for the WHO African region [8]. The framework was structured to address different audiences from the 47 countries of the region, who all provide data, including the Ministries of Health at the centre of effective M&E processes with support from the WHO Country Offices (WCOs), WHO AFRO and international partners.

Here we discuss the processes that led to the development of this framework, its content and implementation, preliminary findings, and challenges and lessons learned. We conclude with forward-looking actions required to strengthen the M&E of COVID-19 and other public health emergencies of potential international concern.

Developing the framework required the following key steps: (i) development of the foundational SPRP for the COVID-19 pandemic; (ii) establishment of a regional multidisciplinary task force responsible for overseeing the development of the M&E framework and its implementation; (iii) development of standard operating procedures and an action plan for implementation of the framework at country level; (iv) development of an electronic information management system to support collection, analysis, interpretation and reporting on progress made in monitoring and evaluating the SPRP; and (v) the overall implementation of the plan of action of the framework. A regional multidisciplinary task force was immediately established as COVID-19 cases were confirmed in the African region, drawn from expertise within and outside WHO AFRO, including representatives from five WCOs and from each of the 11 pillars of the SPRP ((i) coordination, planning and monitoring; (ii) risk communication and community engagement; (iii) surveillance, rapid response teams and case investigation; (iv) points of entry; (v) laboratory services; (vi) infection prevention and control; (vii) case management; (viii) operational logistics and support; (ix) external communication; (x) research, innovation and vaccines; and (xi) continuity of essential health services), M&E experts with previous experience working with UNAIDS, USAID, Global Fund, GIZ, the World Bank and PEPFAR, experts in strategic planning, and data scientists.

This task force used these 11 SPRP response pillars as the basis for defining a set of inputs, outputs, outcomes and impact indicators that were used to closely monitor and evaluate progress in COVID-19 response. Each pillar was tailored to specific country needs and built on the three-pronged preparedness and response strategy [3], (i) coordination and support, (ii) scaling up country readiness and response operations, and (iii) research and innovation. A set of country-specific and regional technical and managerial indicators were developed and critically reviewed to ensure that they provided information that informed action, at the same time creating transparency and accountability.

Criteria outlined by MEASURE Evaluation [9] were applied to crosscheck the robustness of indicators and narrow their scope, based on eight principles: (i) relevance, (ii) accuracy, (iii) importance, (iv) usefulness, (v) feasibility, (vi) credibility, (vii) validity and (viii) distinctiveness [9]. The indicators were assessed by the multidisciplinary team on a scale of one, two or three; three indicating the highest importance.

The selected indicators were further categorised into country-level and regional-level. Country-level indicators cover multisectoral response at the national level (49 indicators across the 11 pillars). The regional-level indicators were used as tools to aid Ministries of Health, WCOs and WHO Regional Offices (59 indicators across the 11 pillars). Together, these repositories provided comprehensive indicators to enable the intended users to monitor, evaluate and understand how the COVID-19 outbreak has been managed at national and regional levels.

Given the rapid spread of COVID-19 in the region, the need for reducing the burden of data collection, and accelerating the process of decision making to adjust the needed public health and social measures to interrupt transmission as the pandemic evolves, a set of 31 key performance indicators (KPIs) were derived from the repositories to facilitate daily and weekly monitoring of the evolution of the pandemic at country and regional levels. These standardised KPIs were selected for reporting to WHO AFRO and aligned with the global M&E Framework in response to COVID-19 in the African Region. Further, a traffic light approach was used to assess each KPI over time, with pre-determined thresholds (Supplementary material S1).

Following the finalisation of the framework, the M&E task force developed a detailed implementation plan. This covered the implementation of M&E activities through immediate and short-term goals (through the end of 2020), medium-term goals (first quarter 2021), longer term goals (through World Health Assembly (WHA), May 2021) and beyond (through 2021). The roll-out of this implementation plan throughout the 47 countries in the region provided guidance on the elements of M&E, the organisation, monitoring and evaluation dimensions, the prioritisation of activities over time, proposed timelines, and estimated resources to support the implementation of the COVID-19 M&E framework. At the country level, WHO AFRO supported the establishment and development of multisectoral task forces, along with partners, to track and coordinate response actions to ensure accountability, as the implementation of country-level M&E for COVID-19 required multisectoral support.

An electronic system was developed to track KPIs through an online portal. Initial data submission was through a standardised Microsoft Excel template, which was subsequently replaced by a web-based database build with OpenDataKit (ODK) and Enketo Smart Paper (https://enketo.org/). Data submission, as well as editing and revising submissions with new or corrected information, was at country level through the WCOs and at regional level through the response pillars. Some regional KPIs were automatically calculated using the country-level data entered, while others required manual entry by each response pillar. The data entry interval was determined by the KPI and could be daily, weekly, monthly and annual at both levels. An online interactive dashboard created with Microsoft Power BI was provided for data entry. This portal is available to the public.

Training was conducted virtually across the region, with six modules introducing the M&E Framework for COVID-19, including full question and answer sessions to solicit feedback from the country offices.

The implementation of this M&E framework provided countries with the structure required to design their own preparedness and response plans early in the COVID-19 pandemic, which were aligned to the global SPRP and thus included an M&E component. As of 8 March 2020, 33 countries had submitted detailed plans to WHO AFRO, with an estimated overall budget of US$166.6 million, the highest from Ethiopia and lowest from Mauritius. Of the nine pillars identified as the priority for the COVID-19 response, logistics required the largest budget, but only 9% of these countries (*n* = 3) submitted plans with budgets that covered all nine pillars. These 33 countries have activated coordination mechanisms and structures to oversee their national pandemic response, including data management and progress reports through daily or weekly situation reports. Twenty-six countries have submitted daily and 15 have submitted weekly situation reports since the start of 2021, while 45 countries submitted regular situation reports through 2020, aligned with guidance provided by WHO AFRO. These, and their associated datasets, are compiled and analysed at WHO AFRO and provide a regional overview of the pandemic.

Thirty-five countries submitted regular line lists of confirmed cases and aggregated data as of 31 January 2021. Three (Côte d'Ivoire, Sao Tome, and Principe and Niger) submitted almost daily; the rest shared weekly or monthly. These line lists were used to compile 31 country profiles, develop detailed epidemiological reports for 28 countries, prepare 108 modelling reports, as well as 34 external situation reports and 14 bi-weekly progress reports on COVID-19 from 5 July to 31 December 2020.

Selected KPIs were used to monitor country progress through a bi-weekly progress scoring tool developed against 10 select KPIs. These KPIs were used to identify priority countries in need of additional support from WHO AFRO. Key indicators included the per cent change in new cases over the previous 2-week period, the case fatality ratio over the last 14 days, number of deaths over the last 14 days, doubling time, testing capacity, number and percentage of healthcare worker infections, recovery rates and attack rates. Assessed on a scale of 0–2 (values were assigned using the traffic light system by pre-determined thresholds), the scores for each of the 10 select KPIs to monitor progress were tallied to provide an overall progress score. The situation was classified as improving if the overall score fell between 0 and 5, stable if the overall score fell between 6 and 10, and deteriorating if the overall score fell between 11 and 20. Following scoring, the 10 indicators for each country were assessed to make recommendations to WHO AFRO regarding immediate priorities. Table 1 shows the number of countries in the WHO African region by progress score for three of the selected KPIs used to monitor country progress over time.

**Table 1.** Number of countries in the WHO African region by score for three selected KPIs used to monitor country progress over time for the last four epidemiological weeks of 2020

Progress indicators for COVID-19 response	Target ranges	Epi week 49	Epi week 50	Epi week 51	Epi week 52	Target
% change in no. new cases compared to previous week (*n* = 46^a^)	⩽−50%	0	7	6	4	A reduction of over 50% during the last 4 weeks corresponds to a sustainable decrease
	−50 to 20%	7	19	22	26	
	>20%	39	20	18	16	
Weekly case fatality rate (CFR) (*n* = 46^a^)	⩽1	27	28	30	29	Target: weekly CFR <1
	1–2.5	15	11	12	13	
	>2.5	4	7	4	4	
% change in no. of health worker infections compared to previous week (*n* = 47)	⩽−50%	1	3	2	6	A decline of at least 50% in the number of new deaths during the last 3 weeks correspond to a durable drop
	−50 to 20%	39	40	40	39	
	>20%	7	4	5	2	

a Tanzania not assessed.

Reviewing existing capacities in M&E of outbreaks and other public health emergencies in countries highlighted the challenges confronted by each of the 47 countries and WHO AFRO, not only in rapidly adapting existing strategic information systems, but most importantly in using M&E systems to routinely track the evolution of the COVID-19 pandemic. Additionally, the limited information available to be used to guide decisions on public health and social interventions became clear. While significant investments have been made over the past years in strengthening the M&E of programmes for HIV, TB and malaria with the support of the Global Fund, World Bank, USAID, PEPFAR and others, the investments in M&E for managing emerging or recurrent outbreaks in the WHO African region were limited. This was clear from the absence of dedicated M&E officers within disease surveillance and response programmes. This likely contributed to the multiplicity of data reporting and requests received from countries, which overburdened WCOs and may have resulted in inconsistencies within the data that originated from different sources. Further, the massive scale of the COVID-19 pandemic and necessary response overstretched the existing limited workforce.

Despite the limited investment in the M&E of health emergencies, the assessment of IHR core capacities in 44 out of 47 countries, the field presence of WHO working closely with Ministries of Health and in-country partners, the adoption of IDSR, and capacity building of disease surveillance officers have resulted in countries systematically activating epidemic management committees, notifying WHO AFRO of any acute public health events, and sharing situation reports and datasets on new and ongoing public health events. These existing systems and procedures were instrumental in collecting, compiling, analysing and sharing data on COVID-19 in the region. The use of electronic systems for evidence-based preparedness and response to COVID-19 contributed to close monitoring of the pandemic in each country and in the region [10].

These indicators were also used to report on progress made by each country and by the region in preparing for and responding to COVID-19. Using selected KPIs for bi-weekly monitoring of country progress, such as cumulative incidence, case fatality ratio, healthcare worker infections and others, facilitated the categorisation and prioritisation of countries in need of immediate support from WHO AFRO and partners. For example, countries with a high percentage of healthcare worker (HCW) infections were supported in strengthening infection and prevention control. Table 1 shows select KPIs used for monitoring country progress and reports the scores for these indicators over the last four epidemiological weeks of 2020, where the number of countries with >20% increase in HCW infections declined from 7 to 2 (Table 1). Further, while initial laboratory capacity presented a challenge in effectively testing for SARS-CoV-2, WHO AFRO used the country progress monitoring to identify countries that needed additional assistance in scaling up their testing capacity. By July 2020, 12 of the 47 Member States reached the target threshold of a weekly average of 10 tests conducted per 10 000 people. By 21 October 2020, a total of 12351482 SARS-CoV-2 PCR tests had been performed in the region.

Routinely measuring progress made by countries in their national response to COVID-19 remains a challenge for several reasons. First, the urgency of supporting the implementation of response interventions takes precedence over the need for carrying out the evaluation of response at national and regional levels. Second, the absence of comprehensive guidance and universal evaluation for COVID-19 national response has led to the proliferation of metrics used to measure progress and evaluate overall response, with less of a focus on subnational assessments. Third, the difficulties in combining public health data with data from other sectors and performing real-time analysis of multiple and often incongruent data have impeded data-driven decision-making processes at different levels.

The importance of a robust M&E framework for COVID-19 cannot be understated. When decision-making during a pandemic needs to be data-driven, M&E plays a crucial role in assessing the continued appropriateness of ongoing response measures and identifying changes that need to be made. Building on the process that led to the development of the framework for the M&E of COVID-19 and its implementation, there is an urgent need for WHO AFRO and the global health community to invest in a versatile M&E framework that can be easily and rapidly adapted to all infectious diseases with a potential for international spread. A comprehensive package of guidelines for M&E of health emergencies that build on the work and lessons learned in dealing with HIV, TB, malaria, EVD and now COVID-19 is critical, with context- or event-specific indicators based on the type of health emergency. The development of such a versatile M&E framework would allow for a more robust M&E process earlier on in the response to future health emergencies.

The COVID-19 pandemic has also demonstrated the importance of a truly multisectoral response, highlighting the close link between public health and economic outcomes. The lessons learned from the experience of developing and implementing the M&E framework for COVID-19 should build on what has been learned from M&E for infectious disease health emergencies such as HIV and EVD. Specifically, while an ultimate end goal may be publicly available data and information systems that combine public health data and data from other sectors, with customisability depending on location-specific needs, a more immediate working priority is the democratisation of data, including the derived analytics and insights. Given the challenges of creating a combined dataset when the required data differ in origin and availability, primary datasets across sectors should be made readily and regularly available to allow for analyses that guide decision-making. Health emergencies such as COVID-19 are rapidly changing, and evidence-based decision-making requires access to up-to-date data.

Additionally, linkage of the M&E systems with research entities focused on areas spanning the multisectoral response will directly inform areas for improved communication and collaboration. A dedicated task force overseeing M&E implementation is critical, as the engagement of national authorities and partners at national and regional levels as part of an all-inclusive process is key to ensuring successful implementation of the framework. Moreover, there is a need for agreement on pandemic performance metrics and indicator selection to standardise M&E assessments across the region, along with a need to build expertise and capacity in data collection, analysis and interpretation, as this is essential for real-time M&E activities. As the pandemic unfolds there is also a strong need for evaluation capacity and a continued need to document the implementation process. Lastly, investment in M&E of health emergencies should be an integral part of efforts to strengthen national, regional and global capacities for early detection and response to threats to public health security.

In conclusion, one year on from the virus's arrival in the African region [10], the COVID-19 pandemic continues to unfold, as new variants and vaccines have entered the picture, emphasizing the continuous learning opportunities available through M&E. While the WHO African region is adept at responding to simultaneous outbreaks and emergencies, the COVID-19 pandemic represented the first time that all Member States were affected simultaneously by the same emergency, highlighting the need for M&E systems that are easily deployed, easy to use, and informative at the national level. As the M&E Framework for COVID-19 built on the lessons learned from EVD and HIV, the development of an adaptable M&E framework for health emergencies must draw from the lessons learned throughout the COVID-19 response.

## Data Availability

The data that support the findings of this study are available on request from the corresponding author [BI]. Some of the data are publicly available through situation reports produced by the Ministries of Health and WHO AFRO on their respective websites, however not all data are publicly available due to confidentiality concerns.

## Supplementary material

For supplementary material accompanying this paper visit https://doi.org/10.1017/S0950268821000807. click here to view supplementary material
