# Supplementary material for: Monitoring and evaluation of COVID-19 response in the WHO African region: challenges and lessons learned
Source: Epidemiol Infect. 2021 Apr 14;149:e98. doi: 10.1017/S0950268821000807 (PMC8723986; doi:10.1017/S0950268821000807)
Supplement: Supplementary file 1 [file S0950268821000807sup001.docx]

**Supplemental Information (S1):** List of 31 key performance indicators (KPIs) for the monitoring and evaluation of the response to COVID-19

| **Coordination & Incident Management** | | | |
| --- | --- | --- | --- |
| **Performance Indicator 1:** % of approved national budget utilized to date | | | |
| **Performance Indicator 2:** % of WCO response funding utilized to date | | | |
| **Performance Indicator 3:** % coverage of IMST staffing need | | | |
| **Performance Indicator 4:** % of national staff in IMST | | | |
| **Performance Indicator 5:** % of WCO IMST staff deployed to or supporting decentralized IMSTs | | | |
| **Control at point of entries** | | | |
| **Performance Indicator 6:** % of travelers who tested positive on arrival in the last 7 days | | | |
| **Performance Indicator 7:** Percentage of designated points of entry with screening, isolation facilities and referral system for COVID-19 | | | |
| **Performance Indicator 8:** Mechanism of tracking travelers from affected countries is in place and operational | | | |
| **Surveillance & control of transmission** | | | |
| **Performance Indicator 9:** % of new confirmed cases among known contacts | | | |
| **Performance Indicator 10:** % of alerts investigated within 24 hours during the last 7 days | | | |
| **Performance Indicator 11:** % of contacts under follow-up seen during the last 24 hours | | | |
| **Laboratory services: testing strategy in place and applied** | | | |
| **Performance Indicator 12:** % laboratory results made available within 48 hours | | | |
| **Performance Indicator 13:** % increase in lab testing capacity | | | |
| **Laboratory services: diagnostic capacity at decentralized levels established and functioning** | | | |
| **Performance Indicator 14:** % new tests performed during the current week by labs at decentralized level | | | |
| **Case Management and IPC** | | | |
| **Performance Indicator 15:** Bed occupancy rate for suspected cases (%) at present | | | |
| **Performance Indicator 16:** Percentage of COVID-19 treatment centers functional at sub-national level | | | |
| **Performance Indicator 17:** Bed occupancy rate for confirmed cases at present | | | |
| **Performance Indicator 18:** Bed occupancy rate for critical and severe COVID-19 cases at present | | | |
| **Performance Indicator 19:** Case fatality ratio | | | |
| **Performance Indicator 20:** % of confirmed cases among healthcare workers | | | |
| **Performance Indicator 21:** % of new confirmed cases among healthcare workers during the last 7 days | | | |
| **Performance Indicator 22:** % of districts that have reported at least one confirmed case during the last 7 days | | | |
| **Performance Indicator 23:** Case fatality ratio of confirmed cases reported during the last 7 days | | | |
| **Performance Indicator 24:** % of new confirmed cases isolated within one day after symptoms onset during the last 7 days | | | |
| **Performance Indicator 25:** % of health care workers trained in case management of COVID-19 cases | | | |
| **Risk communication and community engagement** | | | |
| **Performance Indicator 26:** A rumor management mechanism is in place and operational and evidenced by a report | | | |
| **Safe essential service delivery** | | | |
| **Performance Indicator 27:** % of change in consultations in selected primary health facilities and prenatal clinics | | | |
| **Performance Indicator 28:** % of change in surviving infants receiving third dose of DPT-containing vaccine | | | |
| **Performance Indicator 29:** % of change in ODP attendance | | | |
| **Performance Indicator 30:** % of change in number of people living with HIV in target area who received ART | | | |
| **Procurement of Critical Supplies** | | | |
| **Performance Indicator 31:** Has the WCO experienced any stockouts of critical supplies or essential materials in the last week? | | | |
| **Performance Indicator Assessment** | | | |
| Good |  | 90%-100% | <5% |
| Acceptable |  | 80%-89% | 5-10% |
| Poor |  | Less than 80% | >10% |
|  | | *Indicators 7, 9-11, 15, 17, 18, 24* | *Indicators 6, 19-21* |
|  | | | |
| Good |  | 60%-100% | > 40% |
| Acceptable |  | 40%-60% | 20%-40% |
| Poor |  | Less than 40% | Less than 20% |
|  | | *Indicators 12, 13* | *Indicators 14, 16* |
|  | | | |
| Good |  | >= 0% | No |
| Acceptable |  | (-1%)-(-5%) | -- |
| Poor |  | Less than (-5%) | Yes |
|  | | *Indicators 27-30* | *Indicator 31* |
